# Assessing Osteoporosis in the Young Adult

**DOI:** 10.17925/EE.2015.11.01.43

**Published:** 2015-04-11

**Authors:** Wong Sze Choong, Stephen Gallacher, Syed Faisal Ahmed

**Affiliations:** 1. Honorary Consultant Paediatric Endocrinology, Developmental Endocrinology Research Group, School of Medicine, University of Glasgow, Glasgow; 2. Consultant Physician and Endocrinologist, Diabetes Centre, Southern General Hospital, Glasgow; 3. Consultant Paediatric Endocrinologist, Developmental Endocrinology Research Group, School of Medicine, University of Glasgow, Glasgow, UK

**Keywords:** Osteoporosis, young adult, chronic disease, dual energy absorptiometry, peripheral quantitative computer tomography, magnetic resonance imaging, bone biopsy

## Abstract

Osteoporosis in the young adult is a relatively rare phenomenon, and its diagnosis needs careful assessment of the affected person. The emphasis in the assessment of bone health is gradually shifting from a simple quantitative assessment of bone mineral density to one that includes bone quality. This may be particularly important in the young adult, where the aetiological cause of osteoporosis may be a primary genetic condition or secondary to another chronic condition.

Fragility fractures in the young individual are an uncommon clinical scenario, and when faced with such a situation, appropriate assessment is required to ensure correct diagnosis of the underlying aetiology and to avoid unnecessary interventions. Although primary causes of osteoporosis, such as osteogenesis imperfecta, are considered rare, this group of conditions has considerable phenotypic and genotypic heterogeneity and may be underdiagnosed.^1^ However, most young people with fragility fractures have a secondary cause as the underlying aetiology. This may include a range of chronic diseases and medications that can affect bone turnover, modelling or bone mineral homeostasis.^2^ In light of the increasing prevalence of young adults with childhood-onset chronic disease, it is likely that more people with such conditions will require an assessment of bone health in early adulthood. The diagnosis of osteoporosis in the young adult remains contentious. In growing children, interpretation of results and changes need to take into account the differences in stature, growth and pubertal development, as well as the poor evidence that exists in this population for dual-energy absorptiometry bone mineral density (DXA BMD) as a predictor of fractures.^3^ The International Society of Clinical Densitometry (ISCD) recommends that growing children and adolescents (5–19 years) should not be diagnosed with osteoporosis solely based on low BMD by DXA, with a greater focus on the presence of pathological fractures, such that the diagnosis of vertebral fractures alone is indicative of osteoporosis in the younger individual regardless of DXA parameters.^4^ A stronger emphasis on vertebral morphometry is thus, becoming more routine in the assessment of osteoporosis. With technological advances, it is likely that identification of bone deficits will move away from techniques such as DXA to those that can provide more integrated, yet quantitative, assessment of bone quality through non-invasive techniques, such as high-resolution magnetic resonance imaging (MRI) or computed tomography (CT)^5–7^ or peripheral computer tomography (pQCT), such as microindentation, which can be performed in the clinic setting.^8^

A careful history for systemic symptoms suggestive of an underlying chronic disease or endocrine disorder is crucial. Information on fracture history, including age of fractures, mechanism of injury and review of bone imaging, should be performed, too. Family history, menstrual pattern in females, dieting, exercise and medication history (including over-the-counter medication) and alcohol consumption should be obtained. Physical examination should include assessment of height and weight and pubertal assessment, especially in adolescents and young adults in their 20s. Testicular examination is important, as men with Klinefelter Syndrome may not have the classic body habitus but will invariably have relatively small testes. Deformities at site of previous fractures, hypermobility and blue sclerae, in some, may be suggestive of osteogenesis imperfecta. Laboratory investigations should be aimed at identifying an underlying medical or genetic condition that may lead to the skeletal abnormality. Elevated bone turnover markers are often seen acutely after a fracture, and their reference ranges are very wide to be clinically helpful for initial diagnostic assessment, but low alkaline phosphatase (ALP) may be an indicator of hypophosphtasia. DXA BMD z scores should be assessed in individuals younger than 25 years. In growing adolescents as well as those whose height are outwith the normal range, adjustment for size is also essential. Evaluation of BMD, bone geometry and bone stiffness may also be considered using MRI, CT and microindentation, but these techniques require further validation. In a small subset of individuals, dynamic bone histomorphometry by a transiliac crest biopsy is indicated, especially when the aetiology is unclear in situations such as idiopathic juvenile osteoporosis.^9,10^ Understanding the aetiology in such cases of osteoporosis may also improve the rationale for the use of bone anabolic therapy as opposed to anti-resorptive therapy.^11^

In conclusion, osteoporosis in the young adult that is associated with a fragility fracture is relatively rare and needs careful assessment for an underlying aetiology before treatment is initiated. With technological advances, it is likely that a qualitative assessment of bone will increasingly become incorporated into clinical practice.

## References

[1] Stagi S., Cavalli L., Seminara S. (2014). The ever-expanding conundrum of primary osteoporosis: aetiopathogenesis, diagnosis, and treatment. Ital J Pediatr.

[2] Ahmed S. F., Elmantaser M. (2009). Secondary osteoporosis. Endocr Dev.

[3] Wong S. C., Khanna S., Rashid R. (2008). Auditing bone densitometry and fractures in children with chronic disease. Arch Dis Child.

[4] Gordon C. M., Leonard M. B., Zemel B. S. (2014). 2013 Pediatric Position Development Conference: executive summary and reflections. J Clin Densitom.

[5] Link T. M. (2012). Osteoporosis imaging: state of the art and advanced imaging. Radiology.

[6] McComb C., Harpur A., Yacoubian C. (2014). MRl-based abnormalities in young adults at risk of adverse bone health due to childhood-onset metabolic and endocrine conditions. Clin Endocrinol (Oxf).

[7] Abdalrahaman N., McComb C., Foster J. (2015). Deficits in trabecular bone microarchitecture in young women with type 1 diabetes mellitus. J Bone Miner Res.

[8] Farr J. N., Drake M. T., Amin S. (2014). *In vivo* assessment of bone quality in postmenopausal women with type 2 diabetes. J Bone Miner Res.

[9] Mäyränpää M. K., Tamminen I. S., Kröger H. (2011). Bone biopsy findings and correlation with clinical, radiological, and biochemical parameters in children with fractures. J Bone Miner Res.

[10] Laine C. M., Koltin D., Susic M. (2012). Primary osteoporosis without features of OI in children and adolescents clinical and genetic characteristics. Am J Med Genet A.

[11] Nishiyama K. K., Cohen A., Young P. (2014). Teriparatide increases strength of the peripheral skeleton in premenopausal women with idiopathic osteoporosis: a pilot HR-pQCT study. J Clin Endocrinol Metab.

